# Hypodermic Injection of Quinine

**Published:** 1882-04

**Authors:** J. B. Scriven

**Affiliations:** Late Civil Surgeon of Lahore


THE HYPODERMIC INJECTION OF QUININE. By J. B. SCRIVEN, late Civil Surgeon of Lahore.

The exceptional severity of certain epidemics of malarious fever, such as that which has recently desolated Umritsur, and the frequent difficulty, from the presence of both vomiting and diarrhœa, of administering quinine either by mouth or rectum, naturally suggests the inquiry whether there is not some other mode of introducing the antidote into the system, which shall not be interfered with by the irritability of either stomach or intestine.

I made some remarks on the hypodermic method in a former number of the *Lancet*, and gave a formula for dissolving the quinine with tartartic acid. Having had now four years further experience in the East, I can speak still more strongly of the efficacy and safety of this method of treatment. I have used it in a great number of cases since 1876, being careful to carry out my own proposal for the avoidance of abscess and ulceration—viz., to introduce the nozzle of the syringe so that the aperture, by which the quinine escapes into the cellular tissue, shall be turned *away* from the skin. The result has been that I have not had a single instance of abscess, ulceration, sloughing, or evil effect of any kind, save a temporary inflammation of arm, requiring rest and cold lotions. As a possible exception to this, I ought to mention that one patient, on whom I operated several times, has now a slight contraction of the extensor indicis tendon; this, however, did not arise until some months after the last injection, and she had rheumatism of the wrist in the interval. It is well, from the moment of the injection, to give the limb absolute rest for a few days, in order to limit or prevent the inflammation.

I have had no case of tetanus since I began to use the tartartic solution in 1872; and I believe the dangers, which have led to the prohibition of the subcutaneous use of quinine in the army of India, have no existence, if the precautions I have recommended be observed.

It appears to me that the tartartic solution is greatly preferable to the aqueous solution of the neutral sulphate, on account of its higher concentration, the tartartic solution containing one in three, whereas the solubility of the neutral sulphate is only one in twelve.

In 1876 I made a strong point of filtering the solution. Latterly, finding my quinine free from visibly insoluble particles, I have dispensed with this precaution, as a large quantity of the solution was always absorbed by the filtering paper.—*Lancet.*

H. D. V.

---

### PREVENTION OF THE INTRODUCTION OF CONTAGIOUS DISEASES.

At a special meeting of the Michigan State Board of Health, held at Ann Arbor, March 1, 1882, the following preamble and resolutions were adopted :

WHEREAS, Measures for the prevention of the introduction of diseases from foreign countries into the United States are of national importance, affecting not only the Seaboard and Gulf States, but also States in the interior, as evidenced a few years since by the wide-spread disaster from yellow fever, and recently by the wide diffusion of imported small-pox ; therefore

*Resolved*, That in the judgment of this Board, such measures should be continued by the National Board of Health and undertaken by the United States Government as will best and most effectually prevent the introduction of disease into the United States.

*Resolved*, That our Senators and Representatives in Congress be, and they hereby are respectfully and earnestly, requested to use their influence toward securing any appropriate legislation which may be necessary to this end.

